# The acute inflammatory response to intranigral α-synuclein differs significantly from intranigral lipopolysaccharide and is exacerbated by peripheral inflammation

**DOI:** 10.1186/1742-2094-8-166

**Published:** 2011-11-28

**Authors:** Yvonne Couch, Lydia Alvarez-Erviti, Nicola R Sibson, Matthew JA Wood, Daniel C Anthony

**Affiliations:** 1 Experimental Neuropathology, Department of Pharmacology, University of Oxford, Oxford, OX1 3QT, UK; 2 Department of Clinical Neuroscience, UCL Institute of Neurology, London, NW3 2PF, UK; 3 Gray Institute for Radiation Oncology and Biology, Department of Oncology, University of Oxford Radiobiology Research Institute, Churchill Hospital, Oxford, OX3 7LJ, UK; 4 Department of Physiology, Anatomy and Genetics, University of Oxford, Oxford, OX1 3QX, UK

**Keywords:** brain, inflammation, α-synuclein, SNCA, cytokine, Parkinson's, chemokine

## Abstract

**Background:**

Activated microglia are a feature of the host response to neurodegeneration in Parkinson's disease (PD) and are thought to contribute to disease progression. Recent evidence suggests that extracellular α-synuclein (eSNCA) may play an important role in the pathogenesis of PD and that this may be mediated by a microglial response.

**Methods:**

We wished to discover whether the host response to eSNCA would be sufficient to induce significant cytokine production. *In vitro *cultured BV-2 microglia were used to determine the basic inflammatory response to eSNCA. *In vivo*, 8-week old Biozzi mice were subjected to a single intranigral injection of either 3 μg SNCA, lipopolysaccharide (LPS) or serum protein (BSA) and allowed to recover for 24 hours. A second cohort of animals were peripherally challenged with LPS (0.5 mg/kg) 6 hours prior to tissue collection. Inflammation was studied by quantitative real-time PCR for a number of pro-inflammatory genes and immunohistochemistry for microglial activation, endothelial activation and cell death.

**Results:**

*In vitro *data showed a robust microglial response to SNCA, including a positive NFĸB response and the production of pro-inflammatory cytokines. Direct injection of SNCA into the substantia nigra resulted in the upregulation of mRNA expression of proinflammatory cytokines, the expression of endothelial markers of inflammation and microglial activation. However, these results were significantly different to those obtained after direct injection of LPS. By contrast, when the animals were injected intracerebrally with SNCA and subsequently challenged with systemic LPS, the level of production of IL-1β in the substantia nigra became comparable to that induced by the direct injection of LPS into the brain. The injection of albumin into the nigra with a peripheral LPS challenge did not provoke the production of a significant inflammatory response. Direct injection of LPS into the substantia nigra also induces cell death in a more robust manner than direct injection of either SNCA or BSA.

**Conclusion:**

These results suggest that the presence of eSNCA protein 'primes' microglia, making them susceptible to environmental proinflammatory challenge. For this reason, we hypothesise that where 'inflammation' contributes to the disease progression in PD, it does so in a punctuate manner (on-off) as a result of systemic events.

## Background

Lewy bodies are intracellular deposits containing the ubiquitous CNS protein α-synuclein (SNCA), and are the pathological hallmark of Parkinson's disease (PD) [1]. However, speculation continues as to the exact role of the protein under both healthy and pathological conditions. Brundin *et al. *have proposed that extracellular SNCA (eSNCA) may be responsible for propagating PD pathology in grafted tissue [2]. The mechanism for the physiological release is debated, but *in vitro *studies have demonstrated that SNCA is secreted by living neurons and enters the surrounding medium [3]. It is also possible that dying neurons release SNCA into the extracellular space. eSNCA is present in measurable quantities in cerebrospinal fluid and plasma of individuals with PD. Various groups have suggested that eSNCA over-stimulates the immune system resulting in a neurotoxic central immune phenotype [4,5]. These studies frequently use *in vitro *techniques or over-expression models where the use of specific vectors may interfere with the immune process [6]. Here we use direct *in vivo *application of SNCA protein to study the inflammatory response.

The inflammatory response described in PD is thought to initially result from the activation of microglia [5]. Traditionally, this has been seen to induce a cascade of proinflammatory cytokines that results in feed-forward immune stimulation and a hyperactive inflammatory response. While proinflammatory cytokines have been shown to be present in both post-mortem brains and the cerebrospinal fluid of PD patients [7-9], it is possible that these inflammatory responses may, *in vivo*, be relatively brief and disguise the true role of microglia in PD. It is possible, in terms of eSNCA, that they play a scavenger-like role, merely clearing debris rather than establishing an inflammatory response on the scale of that seen with more traditional mediators of inflammation.

In order to have a good basis for comparison, we employed an intranigral lipopolysaccharide (LPS) injection as a positive control. Recent PD research has used intranigral injections of LPS as a model of disease [10,11]. What is important about these studies is that they produced a model of dopaminergic cell death caused by a local inflammatory response. However, the inflammation in PD is unlikely to be triggered by the same pathways activated by LPS. To date, little comparison of the *in vivo *inflammatory effects of SNCA and LPS has been made.

The aim of the present study was to determine the immune profile resulting from intranigral eSNCA and further to establish whether this profile differs significantly from that induced by LPS administration. Here, we show that SNCA does generate a significant inflammatory response *in vitro *and *in vivo *when compared to control protein, but the response is far less marked than that seen with LPS. However, we also show that the inflammatory response to eSNCA is greatly enhanced if an animal is also given a systemic injection of LPS. Thus, while the inflammatory profile resulting from stimulation with eSNCA or LPS are considerably different, systemic activation of the immune system can produce a local inflammatory response to eSNCA that is comparable to LPS.

## Methods

### Materials

SNCA peptide (rPeptide, Georgia, USA) was maintained as a stock solution of 6 μg/μl in phosphate-buffered saline (PBS; Invitrogen). Amyloid-β peptide (California Peptide Inc., California, USA) was maintained as a stock solution of 6 μg/μl in 0.1% dimethylsulfoxide/PBS. Bovine serum albumin (BSA; Sigma-Aldrich, Poole, UK) was maintained at a stock solution of 6 μg/μl in PBS. LPS (*E. coli *026:B6, Sigma-Aldrich) was maintained as a stock solution of 10 μg/μl in PBS. Peptide concentrations were chosen based on *in vitro *dose response data (not included). LPS doses, both central and peripheral, were based on those currently used in the literature to produce a robust inflammatory response [11,12].

### Cell culture

BV2 cells (a kind gift from Dr. David Brough, University of Manchester) were maintained in DMEM (GIBCO, Invitrogen, Paisley, UK) with 10% heat-inactivated FCS (GIBCO). Cells were treated with LPS, SNCA or amyloid-β in 12-well plates (1.5 × 10^5 ^cells/well) and supernatant samples were removed at time points up to 48 hours after treatment. Supernatants were analysed for TNFα release by ELISA (R&D Systems, Abingdon, UK) and plates were read using a BioRad Model 680 Microplate Reader (BioRad, Hemel Hempstead, UK). For microscopy, cells were grown on sterile coverslips and fixed in an ice-cold 3:1 acetone:methanol solution prior to mounting with DAPI mounting medium (Vector Laboratories).

### Animals

Adult female ABH-Biozzi mice (6 months) were obtained from Charles River and housed under a standard 12-hour light/dark cycle. Animals were provided with food and water *ad libitum *and all procedures were carried out in accordance with the UK Animals (Scientific Procedures) Act, 1986. Animals were anaesthetized in a 2% isoflurane/oxygen mix (2 L/min) and placed in a stereotactic frame (Stoetling Co., USA) under maintenance anaesthesia (1.5%).

### Intranigral injection of peptide

The skull was exposed and a hole drilled above the position of the substantia nigra pars compacta (SNpc) which lies -2.9 mm anterior, -1.3 mm lateral and -4.1 mm ventral from Bregma [13]. Injections of 0.5 μl of stock solutions were made using a graduated glass capillary tube (Drummond Scientific Company, Broomall, PA, USA) over 5 minutes (0.1 μl/min) followed by 2 minutes of rest, to allow diffusion of the injected material, prior to removal of the needle.

### LPS challenge

A subset of animals were challenged with LPS at 0.5 mg/kg i.p. 18 hours after receiving intranigral injections.

### RNA extraction and cDNA preparation

mRNA was extracted using the RNeasy Mini Kit (Qiagen, Crawley, UK) according to the manufacturer's instructions. Briefly, 10-20 mg of frozen tissue was submerged in 300 μl lysis buffer containing 0.001% β-mercaptoethanol. Tissue samples were homogenised using a motor-driven disposable plastic pestle and the resulting suspension transferred to a Qiashredder Mini Spin^® ^column which was then centrifuged at maximum speed in a microcentrifuge. The resulting lysate was mixed 1:1 with 70% ethanol and centrifuged through an RNeasy Mini Spin^® ^column. The column was washed and treated with DNAse 1 for 15 minutes. The column was washed again to remove any final contaminants and RNA was eluted using RNase-free water. RNA samples were then diluted as necessary in order to input 400 ng total RNA into a 10 μl-reverse-transcription reaction. cDNA was synthesised using a Taqman^® ^Reverse Transcription Reagent Kit (Applied Biosystems, Warrington, UK) as per the manufacturer's instructions.

### Quantitative PCR

RT-PCR assays were performed as previously described [14]. Samples were run against standard curves generated from serially-diluted cDNA from LPS-challenged mouse liver. Primer and probe sets for mouse NFkB, IL-1β, TNFα, TGFβ, COX2 and IL-6 were designed using the Roche universal probe library assay design centre. Samples were analyzed using a Roche Light Cycler 480^® ^(Roche Diagnostics, Welwyn Garden City, UK) and all reagents were used according to manufacturer's instructions. Briefly, gene-specific primers were designed and combined with a FAM/TAMRA labelled hybridization probe. PCR was run according to standard conditions [14]. Analysis was performed using the standard curve to determine reaction efficiency followed by a comparative-cycle-threshold method. Results were expressed as relative expression corrected to the house-keeping gene glyceraldehyde phosphate dehydrogenase (GAPDH).

### Nuclear and cytosolic p65 protein analysis

Fresh tissue was extracted from the injection site and run through the ProteoExtract kit (Merck, Nottingham, UK). Briefly, tissue was mixed with 250 μl Extraction Buffer 1 (including protease inhibitors) and incubated at 4°C for 10 minutes under agitation. Insoluble material was pelleted at 1000 G at 4°C for 10 min and the resulting supernatant, the cytosolic subproteome, was removed and stored. The pellet was mixed with 250 μl Extraction Buffer 2 and incubated for 30 min at 4°C under agitation. The insoluble material was pelleted at 6000 G at 4°C for 10 min. The supernatant, the membrane/organelle subproteome, was removed and the pellet mixed with 125 μl Extraction Buffer 3 (including 1.5 μl Benzonase). Following 10 min of incubation at 4°C the insoluble material was pelleted at 7000 G at 4°C for 10 min and the supernatant, the nuclear fraction, was removed. The final fraction, the cytoskeletal subproteome, was discarded. Western blots were performed on the nuclear and cytosolic subproteomal fractions. Subcellular fractions were analyzed by 1DE western blot probing for p65 (AbCam, UK), using actin (cytosolic) and HCDA1 (nuclear) as housekeeping proteins. Quantification was performed using ImageJ software using BSA injected animals as controls.

### Tissue preparation

Animals were surgically anaesthetised with 0.1 ml pentobarbitone and transcardially perfused with heparinised saline (0.9%) followed by a periodate lysine paraformaldehyde solution (PLP: 2% paraformaldehyde, lysine, periodate and 0.05% glutaraldehyde). Brains were removed, post-fixed in PLP for 4 hours and further fixed in 30% sucrose for > 12 hours. 10 μm-sections were cut on a cryostat (Leica, Bucks, UK) and mounted on gelatine-coated slides.

### Immunohistochemistry

An avidin-biotin-peroxidase method was employed for staining the tissue sections [15]. Antigens were detected using antibodies against Iba-1 (Abcam, Cambridge, UK) to detect activated microglia and ICAM-1 (Abcam) Binding was detected using a biotinylated secondary antibody and an ABC standard kit (Vector Laboratories). Visualization was performed using a 0.05% diaminobenzene hydrochloride solution (DAB; Sigma). ICAM-1 and Iba1 analysis was performed using a light microscope (Nikon Labophot-2, Surrey, UK) fitted with an eyepiece graticule of known area. Vessels were counted in areas of highest density around the site of injection and expressed as number of vessels per mm^2^. TUNEL labelling was performed using a NeuroTacs kit (R&D Systems, Abingdon, UK) as per the manufacturer's instructions and developed using a light microscopy-based method (DAB).

## Results

### SNCA and amyloid-β cause NFĸB subunit p65 to migrate to the nucleus

**Figure 1:**
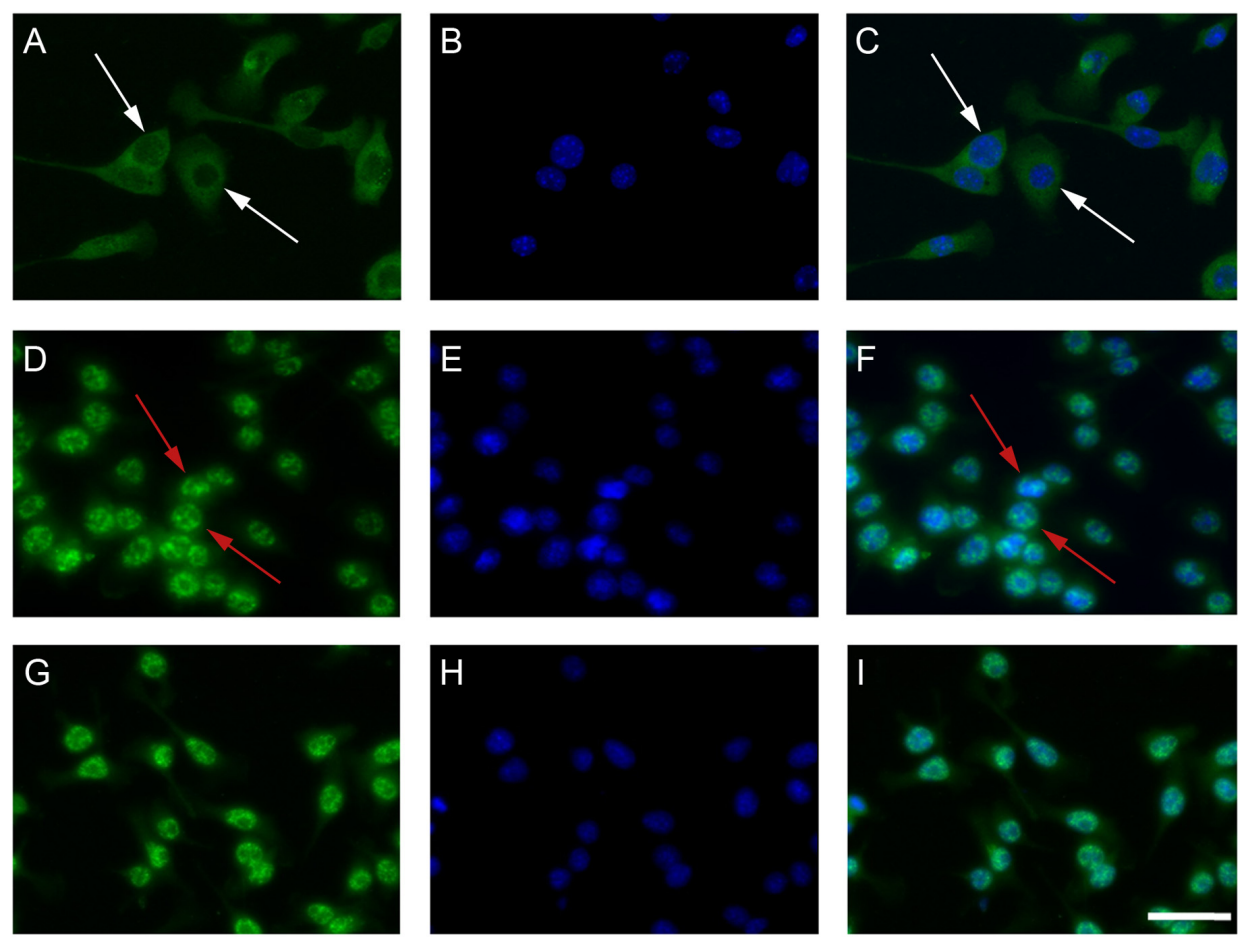
**NFĸB p65 subunit translocation to the nucleus 24 h after treatment with α-synuclein (SNCA) or amyloid-β**. Cells were treated with vehicle (A-C); 3 μg SNCA (D-F) or 3 μg amyloid-β (G-I) for 24 hours at which point cells were fixed and immunostained for the p65 subunit of NFĸB (green; localization indicated by white arrows) and mounted in medium containing the nuclear stain DAPI. Note that SNCA and amyloid-β caused the p65 subunit to translocate to the nucleus (blue; co-localization indicated by red arrows). Scale bar represents 50 μm.

In order to establish whether SNCA and amyloid-β caused changes in inflammatory gene expression via the NFĸB pathway *in vitro *an immortalised murine microglial cell line, BV2, was used. Cells were treated with PBS (Figure 1A-C), 3 μg SNCA (Figure 1D-F), or 3 μg amyloid-β (Figure 1G-I). The cells were fixed with acetone/methanol at 30 minutes, 6 hours and 24 hours. The NFĸB p65 subunit was visualised by immunohistochemistry and the cells were counterstained with DAPI to examine whether the p65 subunit had translocated. From 30 minutes onwards, treatment with SNCA or amyloid-β caused nuclear translocation of the p65 subunit, which was not observed after PBS at any time point. The p65 subunit remained co-localised with the DAPI-stained nucleus 24 hours after the protein treatments. Co-localization analysis from 30 minutes onwards revealed an average Mander's correlation co-efficient of 0.998 in treated cells compared to 0.312 in untreated cells. This indicates a high degree of quantifiable co-localization between DAPI and NFkB in treated groups.

### SNCA, but not amyloid-β, produces significant TNFα release from cultured microglial cells

**Figure 2:**
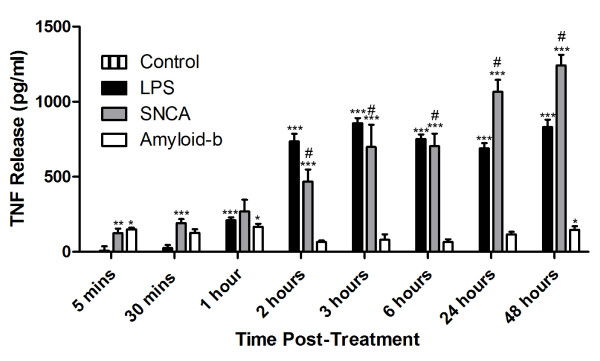
**TNFα release from BV2 cells increases after treatment with LPS and SNCA but not with amyloid-β**. Histogram shows TNFα release (pg/ml) from BV-2 cells as assessed by ELISA at time-points after treatment with vehicle (PBS), 3 μg SNCA, 3 μg amyloid-β or 10 ng LPS. Error bars represent mean ± S.E.M. * represents P < 0.05; ** represents P < 0.01 and *** represents P < 0.001 when compared to control values. # represents significance compared to amyloid-β.

To discover whether SNCA or amyloid-β can be considered proinflammatory *per se*, BV2 cells were incubated with PBS, 3 μg SNCA, 3 μg amyloid-β, or 10 ng (100 EU) LPS as a positive proinflammatory control. Supernatant samples were collected from 5 minutes until 48 hours hours after the application (Figure 2). The level of TNF release was determined by ELISA. TNF production was a feature of all the treatment regimes except PBS. However, despite the similarity in p65 translocation observed after SNCA and amyloid-β treatment, there were clear differences in the extent of TNF release. At 2 hours SNCA produced significantly more TNF than amyloid-β, and the level of TNF continued to rise. TNF produced after SNCA treatment was comparable to that observed with LPS, moreover, by 48 hours SNCA induced more TNF than the LPS treatment. The small initial increase in the level of TNF expression observed after amyloid-β treatment remained unchanged throughout the rest of the time course.

### Direct injection of SNCA into the SNpc upregulates proinflammatory cytokine mRNA

**Figure 3:**
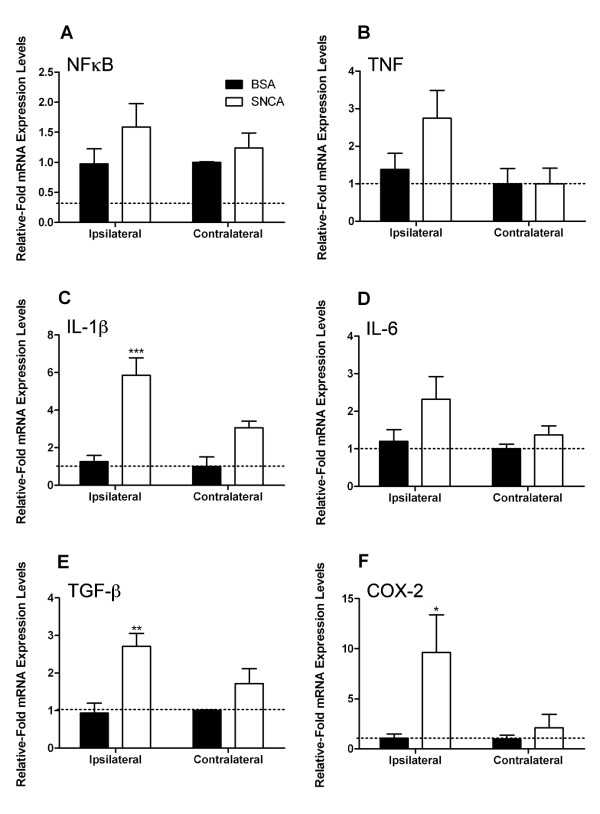
**Cytokine mRNA expression in the brain 24 hours after SNCA or BSA injection into the substantia nigra**. mRNA levels of (A) NFĸB; (B) TNFα; (C) IL-1β; (D) IL-6; (E) TGFβ and (F) COX-2 measured as values relative to GAPDH and normalized to levels within the contralateral hemisphere of control animals. Error bars indicate mean ± SEM. Dotted line represents basal levels in naïve animals. * indicates a significance of P < 0.05 when compared to naïve animals; **indicates P < 0.01 and *** indicates P < 0.005.

As amyloid-β had no significant proinflammatory effects *in vitro *we examined the *in vivo *effects of SNCA, bovine serum albumin (BSA), or LPS administration directly into the SNpc. We found that 24 hours after microinjection of SNCA into the SNpc, the mRNA of the major proinflammatory cytokines, IL-1β, IL-6 and TNFα, were significantly up-regulated compared to the BSA controls (Figure 3). Both eSNCA and BSA failed to alter transcription of NFĸB (p65) (Figure 3A). However, the microinjection of SNCA did result in a 2-fold increase in TNFα gene expression when compared to the contralateral hemisphere of BSA injected animals (Figure 3B). eSNCA produced a significant 5-fold increase in the levels of IL-1β mRNA expression when compared to BSA injected animals (Figure 3C). Similarly, IL-6 is increased after treatment with SNCA compared to BSA animals (Figure 3D). Finally, we found significant increases in TGFβ (2.75-fold) and COX-2 (10-fold) mRNA levels in the ipsilateral hemisphere of SNCA injected animals when compared to the contralateral hemisphere of BSA injected animals (Figure 3E &3F).

### The cytokine profile after LPS injection into the SNpc significantly differs from direct SNCA injection

**Figure 4:**
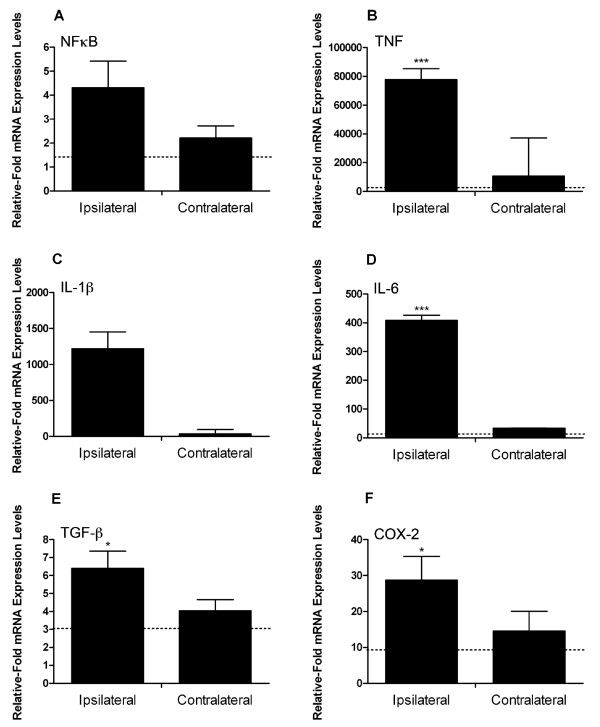
**Cytokine mRNA expression in the brain 24 hours after LPS injection into the substantia nigra**. mRNA levels of (A) NFkB; (B) TNF; (C) IL-1β; (D) IL-6; (E) TGFβ and (F) COX-2 measured as values relative to GAPDH and normalized to levels within the contralateral hemisphere of control animals. Error bars indicate mean ± SEM. Dotted line represents basal levels in naïve animals. * indicates a significance of P < 0.05 when compared to naïve animals and *** indicates P < 0.005.

Direct injection of endotoxin into the SNpc is now frequently used as a model of PD-like neurodegeneration. We aimed to determine whether the inflammatory profile seen with this method differed significantly to that obtained with eSNCA. Microinjection of LPS increased mRNA for NFĸB 4-fold, greater than eSNCA but not significant (Figure 4A). However, microinjection of LPS results in a 77 k-fold increase in TNF mRNA expression (Figure 4B). Increases in IL-1β (1200-fold; Figure 4C) and IL-6 (400-fold; Figure 4D) mRNA expression, measured after LPS microinjection, were significantly higher than the ipsilateral hemisphere of SNCA injected animals. After injecting LPS, mRNA for TGFβ increased 6-fold (Figure 4E), significantly different from the levels recorded in the contralateral hemisphere of BSA injected animals and double those recorded after microinjection of SNCA. A 30-fold (Figure 4F) increase in COX-2 mRNA was observed after central LPS administration, 3-fold higher than the increase seen with eSNCA.

### eSNCA causes significant activation of microglia

**Figure 5:**
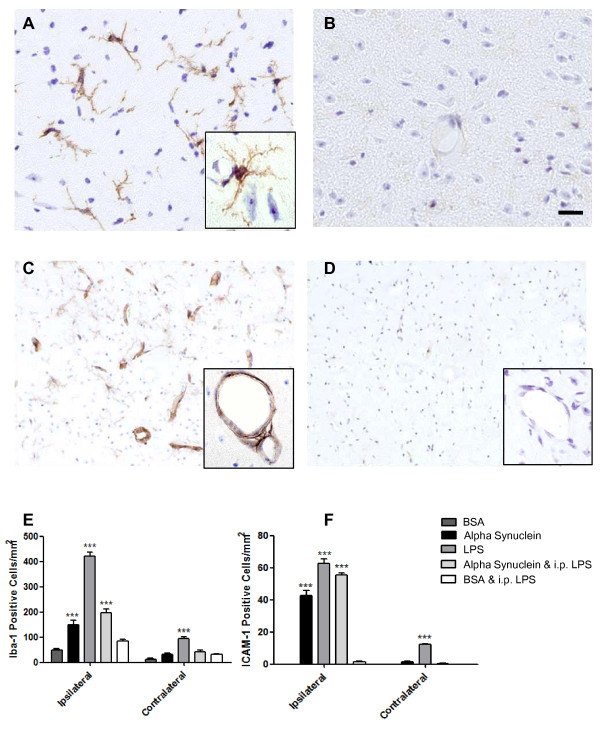
**Increased Iba-1 and ICAM-1 expression 24 hours after intranigral administration of SNCA**. Representative microscopy showing typical Iba-1 (A & B) and ICAM-1 (C & D) staining seen 24 h after injection of SNCA (A & C) or BSA (B & D; both micrographs are counterstained with cresyl violet). Scale bar represents 10 μm. The number of Iba-1-positive microglia (E) or ICAM-1- positive vessels were quantified in both SNCA and BSA injected animals.

Microglial activation was analyzed by immunohistochemistry using an anti Iba-1 antibody, which recognises ionized calcium binding adaptor molecule-1, an EF-hand protein that is expressed by microglia and up-regulated during episodes of inflammation. At 24 hours there was a significant increase in the number of Iba-1-positive cells (Figure 5A) in the ipsilateral hemisphere of SNCA-injected animals when compared to the contralateral hemisphere (Figure 5B). There were also significantly more activated microglia in SNCA-injected animals in the ipsilateral hemisphere when compared to the ipsilateral hemisphere of BSA-injected animals (Figure 5F).

### eSNCA upregulates markers of vascular inflammation

Intercellular adhesion molecule (ICAM) expression after SNCA and BSA treatment was analysed by immunohistochemistry using an anti-ICAM-1 antibody. ICAM is ubiquitously expressed at low concentrations but will increase after exposure to proinflammatory cytokines in order to facilitate leukocyte migration across the endothelium. At 24 hours there was a significant increase in the number of ICAM-1-positive vessels (Figure 5C) in the ipsilateral hemisphere of SNCA-injected animals when compared to the contralateral hemisphere (Figure 5D). There were also significantly more ICAM-1-positive vessels in SNCA-injected animals in the ipsilateral hemisphere when compared to the ipsilateral hemisphere of BSA-injected animals (Figure 5E). Vascular cell adhesion molecule (VCAM) expression was analyzed by immunohistochemistry using an anti-rat VCAM antibody produced in-house (results not shown). Unlike ICAM, VCAM is only expressed during inflammatory episodes when the microvasculature has been exposed to proinflammatory cytokines. Very little VCAM staining was observed in the parenchyma at any time point after any treatment.

### Peripheral LPS exacerbates the proinflammatory potential of eSNCA

**Figure 6:**
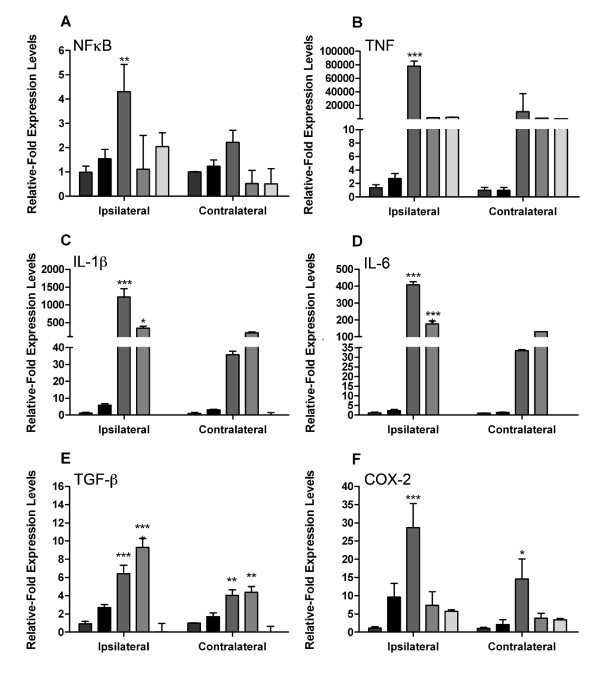
**Cytokine mRNA expression in the brain 24 hours after SNCA, BSA or LPS injection into the substantia nigra with and without peripheral LPS challenge**. mRNA levels of (A) NFkB; (B) TNFα; (C) IL-1β; (D) IL-6; (E) TGFβ and (F) COX-2 measured as values relative to GAPDH and normalized to levels within the contralateral hemisphere of control animals. Bars show mean ± SEM. * indicates a significance of P < 0.05 when compared to non-LPS-injected control animals; **indicates P < 0.01 and *** indicates P < 0.005.

**Figure 7:**
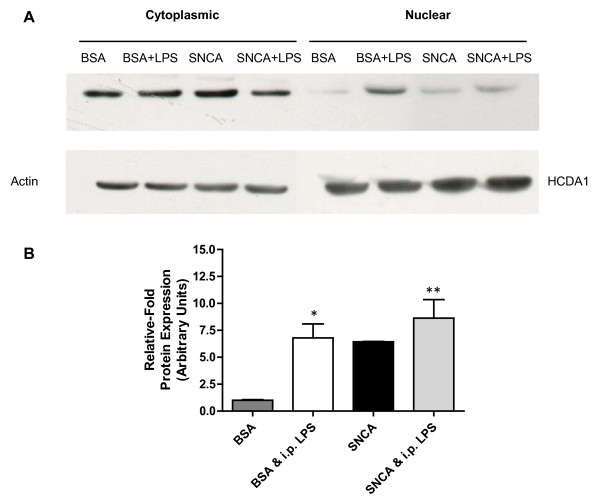
**p65 nuclear translocation in injected hemisphere 24 after BSA or SNCA injection into the substantia nigra with and without peripheral LPS challenge**. (A) p65 protein was visible in both cytosolic and nuclear fractions of brain protein lysate of injected animals. p65 protein levels within the nuclear fraction (B) were measured as relative-fold changes compared to non-LPS injected control animals. * indicates a significance of P < 0.05 when compared to non-LPS injected controls; ** indicates a significance of P < 0.01 when compared to non-LPS SNCA controls.

As systemic infections are known to contribute to the progression of many neurological diseases, we were interested to discover whether the activation of the innate immune system by LPS would affect the host response to eSNCA. Groups of animals were challenged with either systemic LPS or saline at 18 hours after the injection of either SNCA or BSA into the SNpc. At 24 hours, when the animals were killed, TNF was elevated by the systemic LPS challenge in both the ipsilateral and contralateral hemispheres, and the level of expression was independent of the substance injected into SN (Figure 6). However, the levels of IL-6 and IL-1β were significantly elevated by the LPS challenge compared to SNCA controls with no systemic challenge. In the BSA injected animals IL-6 and IL-1 were not significantly different from naïve controls. It is interesting to note that contralaterally IL-6 and IL-1 were also significantly elevated by the systemic LPS treatment to levels that are comparable to the central injection of LPS. COX-2 expression in the SNpc was unaltered by systemic LPS treatment, but TGF-β expression was significantly increased by the LPS challenge in the SCNA-treated animals alone. While NFĸB mRNA expression was unaffected by the challenge, Western blot analysis of protein from the injected hemisphere revealed that p65 nuclear translocation was elevated in SNCA-treated animals, and that a systemic LPS challenge exacerbated this response (Figure 7).

### Central LPS causes cell death

**Figure 8:**
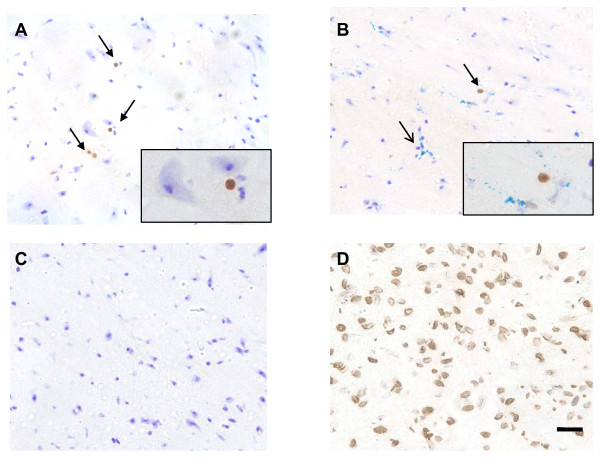
**Cell death in injected hemisphere 24 h after SNCA, BSA or LPS injection into the substantia nigra with and without peripheral LPS challenge**. TUNEL staining was clearly visible in LPS-injected (A) animals but TUNEL was rarely seen in SNCA injected animals (B). No TUNEL staining was observed in BSA-injected brain (C; representative image). Nuclease treated positive control section (D). Data are mean ± SEM, *** indicates a significance of P < 0.005 when compared to the contralateral hemisphere. Scale bar represents 10 μm.

Cell death via apoptosis is an important aspect of a number of neurodegenerative diseases. In order to determine whether the microglia were simply phagocytosing dead cells or actively clearing up damaged ones, it was important to quantify cell death. Using the same groups as previously described nick-end labelling was used to quantify the number of cells undergoing apoptosis at 24 hours post challenge. Only in brains injected directly with LPS were the cells quantifiable (Figure 8D) and clearly visible (Figure 8A). A few isolated TUNNEL-positive cells were observed after SNCA injection, but the number of positive cells was negligible compared to those observed in LPS-injected brains.

## Discussion

The purpose of this study was to investigate the inflammatory properties of SNCA *in vitro *and *in vivo*. Our data reveals that non-aggregated wild-type (wt) synthetic human SNCA produces an inflammatory response *in vitro*, as demonstrated by the translocation of the p65 subunit of NFĸB and the release of TNFα into the surrounding medium, as well as an *in vivo *response. However, to put the qPCR data from SNCA-injected animals into context, a second group of mice received a single intranigral injection of LPS and were allowed to survive for 24 hours prior to tissue processing. The expression levels of proinflammatory mRNA were consistently increased for LPS-injected mice compared to SNCA-injected mice, suggesting that the LPS challenges used to model PD are unlikely to be pathophysiologically relevant. However, when the animals were injected intracerebrally with SNCA and subsequently challenged with systemic LPS the level of production of IL-1β in the SNpc was comparable to that induced by the direct injection of LPS into the brain. The injection of albumin into the SNpc with a peripheral LPS challenge did not provoke the production of IL-1β. Thus, the inflammatory response to eSNCA is context sensitive and suggests the contribution of inflammation induced by eSNCA to the progression of PD is greatly augmented by peripheral immune activation. The significance of these data is discussed below.

NFĸB exists as a heterodimer of p65 and p50, which, in the resting state, has a cytoplasmic distribution. The activation of macrophages by proinflammatory cytokines and by scavenger receptors causes the NFĸB heterodimer to dissociate from its IkB inhibitory accessory subunit and translocate to the nucleus [16]. Our initial findings show that both wt SNCA and wt amyloid-β induce NFĸB-p65-subunit translocation to the nucleus. Others have isolated cytosolic and nuclear fractions of murine microglia and demonstrated the translocation of p65 after one hour of treatment with nitrated SNCA, but no comparison with amyloid-β was performed [17]. While SNCA and amyloid-β both caused translocation of p65, downstream TNF production in response to these challenges was quite different. SNCA gave rise to a rapid and sustained TNF response in BV2 microglial cells, which exceeded the response generated by LPS. Amyloid-β-induced TNF production was trivial by comparison. These results suggest that LPS, SNCA, and amyloid-β activate BV2 cells in a distinct manner. It is also possible that the amyloid-β is cleared more rapidly than SNCA by the BV2 cells. Indeed, results indicate that amyloid-β is rapidly produced and cleared from the CNS in humans and that about 8% of extracellular amyloid-β is turned over each hour [18]. Thus microglial cells are likely to be constantly exposed to amyloid-β, and eSNCA exposure is likely to be more rare. The exposed residues of peptides such as SNCA and amyloid-β may also alter the inflammatory response. Work with the shorter 25-35-residue fragment of amyloid-β is often undertaken because it produces exaggerated toxicological properties when compared to the full-length peptide [19-21]. Indeed, the treatment of astrocytes with full-length amyloid-β has been reported to decrease TNF production when they are co-cultured with monocytes [22]. Reynolds, working on nitrated SNCA, also reported TNF release within the same range as we found here [23]. However, Zhang and colleagues failed to show TNF production by primary mixed neuron-glial cultures with full-length amyloid-β [5]. This might be attributable to differences in the aggregation process. However, together these results highlight the difficulty associated with the interpretation of *in vitro *experiments.

*In vivo*, modest increases in the mRNA of the transcription factor NFĸB were noted in both SNCA- and LPS-treated animals. This was largely expected of a constitutively active, translocating protein. However, it is known that NFĸB proteins in monocytes alter significantly following cellular maturation in culture and we were interested to note whether any of the responses to the *in vivo *challenges would be underpinned by an increase in the overall level of NFĸB signal transduction machinery [24]. The level of NFĸB mRNA was not significantly increased following the peripheral administration of LPS. However, the mechanistic principle of NFĸB activation is nuclear translocation rather than an increase in expression and *in vivo *experiments have shown that p65 translocation to the nucleus is increased in SNCA-treated, and LPS challenged animals.

A 1.25-fold increase in the level of TNF mRNA was observed after SNCA treatment, which is less than that observed in PD brains [17], but similar to that observed in SNCA over-expressing mice [25]. This is likely to represent differences in the length of disease progression and may also be a consequence of the altered reactivity of the aged brain [26] and the presence of environmental challenges or co-morbidity [27]. IL-1β mRNA was increased 5-fold, an increase similar to that measured in SNCA over-expressing mice [25], but considerably smaller than the 1000-fold increase generated by LPS in the SNpc. High levels of IL-1β are a common feature of peripheral inflammatory disease where cell death is a prominent feature [28], but levels of IL-1β are low in the degenerating brain [29]. The principle source of IL-1 in the naïve brain is the microglial cell where it contributes, among others, to the regulation of sleep cycles, appetite and temperature control. IL-6 is also implicated in a number of normal physiological processes within the CNS, including temperature regulation. It is perhaps, therefore, no surprise that IL-1β and IL-6 expression in response to SNCA is limited given the important role of these cytokines in homeostatic processes in the normal brain. However, it is clear that it is possible to overcome any resistance to cytokine production in circumstances where microglial cells have become 'primed' and are then faced with environmental challenges. Here, host survival mechanisms may take precedence over the potential damaging consequences of increased cytokine production in the brain. TNF levels were significantly increased in the ipsilateral and the contralateral hemispheres following the systemic LPS challenge. IL-1β and IL-6 were also increased in the contralateral hemisphere as well as the ipsilateral, but only in those animals that had been injected with SNCA. We have previously noted that distinct cytokine induction pathways do exist in the CNS and that the cytokine network that is often described in the periphery cannot be extrapolated to the brain [30]. The presence of 'mirror' lesions in the contralateral hemisphere of MS patients has been recognised for a long time [31], and, while it is often stated that MS lesions localize in a random fashion, more careful analysis reveals a distinct symmetry when small plaques and all demyelinating activity are taken into account. The signal for contralateral cytokine induction is unknown and it is unclear whether such cross-talk might contribute to PD pathology.

SNCA injection resulted in an increase in TGFβ and COX-2 mRNA but, unlike the proinflammatory cytokines, the levels were not increased by the same order of magnitude after central LPS injection. The values obtained are similar to those reported by others after hippocampal LPS injection [32,33]. TGFβ has been shown to promote neuronal survival in neonatal animals [34] and is often described as an anti-inflammatory cytokine. The pattern observed in this study is similar that observed in murine prion disease [29,32,33]. TGFβ and COX-2 are associated with a non-inflammatory microglial response to neurodegeneration that presumably reflects a phagocytic phenotype, similar to an M2 macrophage, rather than a classically activated M1-like macrophage. The induction of IL-1β and TNF in the SNCA-injected brain, albeit small, suggests that a mixture of phagocytic and inflammatory microglia is generated [35]. Following the central injection of LPS the inflammatory cytokine response dwarfed the observed elevation in TGFβ and COX, and reveals the potential for cytokine production in the brain. It is interesting to note that TGFβ was induced by the combination of peripheral LPS with SNCA to a similar extent as central LPS, but COX-2 was unchanged by LPS with SNCA, which reflects dissociation between the regulatory pathways of these two 'anti-inflammatory' mediators. It should also be noted that SNCA injection did not result in significant neuronal cell death, unlike central LPS injection.

Administration of SNCA provoked a significant inflammatory profile in BV2 cells *in vitro*, which was mimicked *in vivo *with the expression of mRNA for the major proinflammatory cytokines. However, compared the central administration of LPS to the SNpc, cytokine production was only a small fraction of the potential production. Recently LPS has been used as a model of inflammatory PD and while it shows degeneration of tyrosine hydroxylase-positive neurons within the SN, this response is clearly distinct from the microglial response to eSNCA. Initially, such results suggested that the injection of LPS into the SNpc is not a relevant model of PD. However, the peripheral injection of LPS did induce a marked increase in inflammatory activity in the SNCA-injected SNpc. The LPS injections into the SNpc may, therefore, be modeling the consequences of a systemic infection on the degenerating SN. Recent work by Gao and colleagues has substantiated this finding by using a two-hit approach, systemic inflammation in a transgenic model of PD [36]. Transgenic models remain important for studying the long term effects of gene-environment interactions, however, as the authors describe, there are often discrepancies between the progression of the disease in humans and that which occurs in the animal models [37,38]. We sought to study the immediate interactions between SNCA and the immune system and our findings are similar to recent studies in which systemic LPS injections in models of either brain injury or chronic neurodegenerative disease exacerbate brain damage and modify the local inflammatory response [39-42]. For example, a link between MS relapse and infection has been proposed [43]. A causative link between an infective agent and PD has also been sought for some time, but the evidence has been unconvincing. More recently, proponents of the infective hypothesis for idiopathic PD have suggested that certain specific pathogens, such as *H. Pylori*, will be contributory rather than causative, which seems more likely [44]. However, following the studies presented here, it is now our hypothesis that any activator of the innate immune system, including the presence of a pathogen in the periphery, will have the capacity to alter the pathogenesis of PD by generating a transient proinflammatory, destructive microenvironment that is likely to accelerate local neurodegeneration.

## Competing interests

The authors declare that they have no competing interests.

## Authors' contributions

YC and LA-E conducted the experiments and drafted the manuscript. NRS and MJAW assisted with revision of the manuscript. DCA provided intellectual input into experimental design and drafted the manuscript. All authors have read and approved the final manuscript.
